# Lower Urinary Tract and Gastrointestinal Dysfunction Are Common in Early Parkinson's Disease

**DOI:** 10.1155/2020/1694547

**Published:** 2020-10-17

**Authors:** Daniel Martinez-Ramirez, Edna Sophia Velazquez-Avila, Alejandro Almaraz-Espinoza, Arnulfo Gonzalez-Cantú, Genaro Vazquez-Elizondo, Daniel Overa-Posada, Amin Cervantes-Arriaga, Mayela Rodriguez-Violante, Mirna Gonzalez-Gonzalez

**Affiliations:** ^1^Tecnologico de Monterrey, Escuela de Medicina y Ciencias de la Salud, Ave. Morones Prieto 3000, Monterrey 64710, Nuevo Leon, Mexico; ^2^Christus Muguerza Alta Especialidad, Ave. Hidalgo 2525, Monterrey 64060, Nuevo Leon, Mexico; ^3^Oncare Gastrointestinal Services, Calz. San Pedro 255, San Pedro Garza Garcia 66220, Nuevo Leon, Mexico; ^4^Movement Disorders Clinic, National Institute of Neurology and Neurosurgery, Ave. Insurgentes Sur 3877, La Fama 14269, Mexico City, Mexico

## Abstract

**Purpose:**

Autonomic dysfunction is a common nonmotor feature and early manifestation of Parkinsons disease (PD). Autonomic dysfunction in PD is associated with a worse prognosis. We sought to characterize autonomic dysfunction and identify associated factors in patients with early PD.

**Methods:**

An observational, cross-sectional, descriptive, and analytical study was conducted to evaluate patients with early PD from the Parkinsons Progression Markers Initiative. We utilized the Scales for Outcomes in Parkinsons Disease-Autonomic dysfunction questionnaire to determine the prevalence and frequencies of autonomic symptomatology. The cohort was grouped into high and low dysautonomic scores. A regression model identified variables that independently explained dysautonomic scores in our early PD cohort.

**Results:**

414 PD patients had a mean age of 61.1 (SD 9.7) years at diagnosis and mean disease duration of 6.7 (SD 6.6) months. Among all patients, 43.7% (181/414) had high dysautonomic scores. Urinary and gastrointestinal symptoms were the most prevalent and frequently reported dysautonomic symptoms. Patients with fatigue (beta = 4.28, *p* < 0.001), probable rapid eye movement sleep behavior disorder (beta = 2.71, *p* < 0.001), excessive daytime sleepiness (beta = 1.88,*p*=0.039), impulsivity and compulsivity (beta = 2.42, *p* < 0.001), and increasing age (beta = 1.05, *p* < 0.001) were more likely to have high dysautonomic scores.

**Conclusion:**

Lower urinary tract and gastrointestinal symptoms are prevalent and frequent in early PD patients. Fatigue, sleep disorders, impulsivity and compulsivity, and age are predictors of autonomic dysfunction. Autonomic symptoms predominated in this group of early PD patients in the disease course and were associated with more severe disease.

## 1. Introduction

Autonomic dysfunction in patients with Parkinson's disease (PD) was initially reported in the clinical description of PD by James Parkinson in his essay on shaking palsy in 1817 [1]. Research on autonomic dysfunction in patients with PD has increased in the last 30 years [2], and autonomic dysfunction is a well-recognized nonmotor feature of PD caused by peripheral and central autonomic Lewy body accumulation, affecting approximately 40% to 85% of patients during the course of the disease [3, 4]. Autonomic dysfunction may be one of the earliest prodromal manifestations of PD, occurring years or even decades before the appearance of typical motor symptoms [5].

Autonomic dysfunction in PD is typically associated with negative outcomes and has been reported to be correlated with more severe disease [6]. Patients with PD and autonomic dysfunction can experience more hospitalizations, emergency room visits, and telephone calls and e-mails to physicians, higher health-related costs, and shorter survival [7]. Studies assessing autonomic dysfunction in PD have focused mainly on cardiovascular symptoms, and literature on patients with early PD is scarce. Identification of these patients with early PD is crucial for developing therapeutic strategies to prevent complications. Some validated questionnaires and clinical rating scales are useful for easily detecting and characterizing autonomic symptoms in clinical practice [8]. The Scales for Outcomes in Parkinson's Disease-Autonomic dysfunction (SCOPA-AUT) is a clinical questionnaire that assesses the presence and frequency of autonomic symptoms in patients with PD [9]. Since people without PD can also present with autonomic symptoms and higher SCOPA-AUT scores are more frequently associated with neurodegenerative synucleinopathies [10], dichotomizing outcome scores into high and low scores is necessary to help discriminate between symptoms related to PD and symptoms that are not related to PD. Thus, we sought to characterize autonomic dysfunction and identify associated factors in patients with early PD.

In this article, we describe an observational study of patients who were recently diagnosed with PD and underwent evaluation with a clinical autonomic scale and in which we compared demographic and clinical variables in patients with high and low autonomic dysfunction scores.

## 2. Methods

We designed an observational, cross-sectional, descriptive, and analytical study to identify factors associated with the presence and frequency of autonomic dysfunction in patients with early PD. The data were obtained from the Parkinson's Progression Markers Initiative (PPMI), an international observational clinical study that evaluated patients with early PD and controls using advanced imaging, biologic sampling, and clinical and behavioral assessments to identify biomarkers of PD progression [11]. To be eligible for PPMI, patients had to be diagnosed less than two years before enrollment. Of the total 424 patients with de novo early PD who enrolled, 414 patients were included in our study. The remaining 10 patients were excluded due to missing data.

The demographic data obtained from the dataset were age, gender, education level, ethnicity, race, and family history. The following clinical variables were documented: age at symptom onset, age at diagnosis, disease duration, side of symptom onset, Hoehn and Yahr (HY) scale score, Schwab and England Activities of Daily Living (S&E) scale score, and the Movement Disorder Society-Sponsored Revision of the Unified Parkinson's Disease Rating Scale (MDS-UPDRS) score. To evaluate autonomic dysfunction as the outcome variable in the studied cohort, we utilized the SCOPA-AUT questionnaire [9]. The SCOPA-AUT questionnaire is a self-reported questionnaire that evaluates the presence and frequency of dysautonomic symptoms and includes 7 items for gastrointestinal function, 6 items for urinary function, 3 items for cardiovascular function, 4 items for thermoregulatory function, 1 item for pupillomotor function, and 4 items for sexual function (2 for men and 2 for women). Items are scored on a Likert-type scale ranging from 0 (never) to 3 (often). The maximum score is 69, with higher scores indicating more frequent symptoms. We analyzed the questionnaire in two ways. First, we sought for the prevalence of symptoms, for which a score of 0 indicated symptoms were not present and scores from 1 to 3 indicated symptoms were present. Second, we determined the frequency of symptoms, for which a score of 0 indicated there were never symptoms, a score of 1 indicated there were sometimes symptoms, a score of 2 indicated there were regular symptoms, and a score of 3 indicated there were often symptoms. To assure that dysautonomic symptoms were most likely related to PD and not to aging, the SCOPA-AUT scores were divided into two categories. Previous studies have utilized cutoff scores between 9 and 13.1 based on their own criteria [12, 13]. We arbitrarily selected a cutoff of 10 based on the mean (SD) of the total SCOPA-AUT  score of the control patients, who reported a score of 5.8 (3.7). We therefore divided the PD cohort into patients with a SCOPA-AUT score of <10 and those with a score of ≥10.

To evaluate for other nonmotor symptoms, we used the following scales documented in the dataset with PPMI's recommended cutoff scores: the Montreal Cognitive Assessment (MoCA) cutoff of <26, University of Pennsylvania Smell Identification Test (UPSIT) cutoff of <19, Geriatric Depression Scale (GDS) cutoff of ≥5, State-Trait Anxiety Inventory (STAI) cutoff of ≥41, REM Sleep Behavior Disorder Screening Questionnaire (RBDSQ) cutoff of ≥6, and Epworth Sleepiness Scale (ESS) cutoff of ≥10. The Questionnaire for Impulsive-Compulsive Disorders in Parkinson's Disease—Rating Scale (QUIP-RS) results were reported as positive if any impulsive or compulsive symptoms were reported by the patient. The symptoms of apathy and fatigue were documented utilizing the MDS-UPDRS items 1.5 and 1.13, respectively. A cutoff score of ≥2 was chosen to indicate the presence of symptoms.

The mean and standard deviation were used for continuous variables, and frequencies and percentages were used for categorical variables. The distribution of continuous variables was verified using the Kolmogorov–Smirnov test. We employed a chi-squared test to determine associations between independent categorical variables and categorical outcome variables. Student's *t*-test was utilized to determine associations between continuous independent variables and categorical outcome variables. Odds ratios (OR) and Cohen's *d* effect size were calculated to determine the strength of association between variables. We used a backward Wald stepwise elimination to construct a multiple logistic regression model to identify variables that independently explained the SCOPA-AUT scores in our cohort. Only the significantly associated variables from univariate analyses were included. Multicollinearity between variables was tested with a variance inflation factor. We selected the model with less deviance. The IBM Statistical Package for the Social Sciences version 25 was used for the analysis.

## 3. Results

The cohort of 414 PD patients had a mean age of 61.1 (SD 9.7) years at diagnosis and mean disease duration of 6.7 (SD 6.6) months.  Table 1 describes and compares the prevalence and frequencies of SCOPA-AUT domains between high and low SCOPA-AUT score groups. In the total PD cohort, urinary symptoms were the most prevalent and frequent symptoms, followed by gastrointestinal, thermoregulatory, sexual, pupillomotor, and cardiovascular symptoms. A SCOPA-AUT score of ≥10 was observed in 43.7% (181/414) of the PD cohort. When comparing high and low SCOPA-AUT score groups, urinary and gastrointestinal symptoms remain as the most prevalent and frequent symptoms in both groups. Patients with gastrointestinal and urinary symptoms were 11.6 and 17.8 times more likely to report a SCOPA-AUT score of ≥10 than patients without these symptoms, respectively. In addition, individuals with SCOPA-AUT scores ≥10 experienced more frequent gastrointestinal and urinary symptoms than individuals with SCOPA-AUT scores <10. The effect size for both analyses exceeded Cohen's convention for a large effect (*d* = .80), as shown in Table 1.

**Figure 1:**
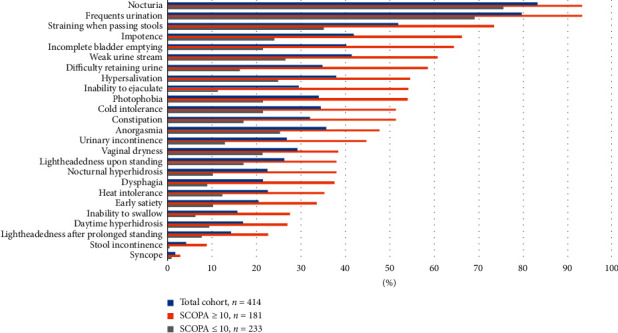
Prevalence of SCOPA-AUT items in our PD cohort. SCOPA-AUT: Scales for Outcomes in Parkinson's Disease-Autonomic dysfunction; PD: Parkinson's disease.

**Figure 2:**
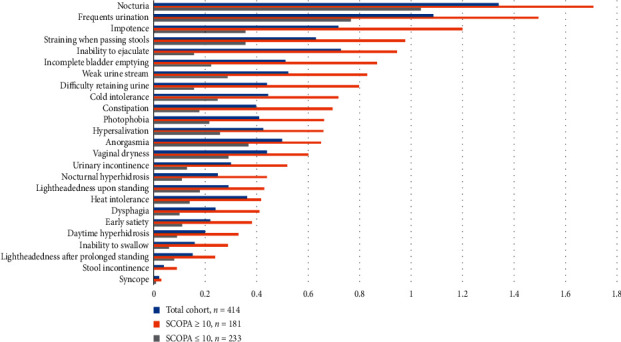
Frequency of reported SCOPA-AUT items in our PD cohort. SCOPA-AUT: Scales for Outcomes in Parkinson's Disease-Autonomic dysfunction; PD: Parkinson's disease.

We separately analyzed each domain of the SCOPA-AUT questionnaire. The most common symptoms reported by the total cohort were the need to strain when passing stools in the gastrointestinal domain, nocturia in the urinary domain, symptoms of orthostatic hypotension in the cardiovascular domain, cold intolerance in the thermoregulatory domain, problems having or maintaining an erection in men and anorgasmia in women in the sexual dysfunction domain, and photophobia in the only pupillomotor domain. The high and low SCOPA-AUT score groups experienced the same three most common symptoms: frequent urination, nocturia, and straining when passing stools. Figures 1 and 2 show the prevalence and frequencies of SCOPA-AUT domains between high and low SCOPA-AUT score groups.

An independent samples *t*-test was conducted to compare the demographic and clinical variables between the SCOPA-AUT score groups (Table 2). There were significant differences between the high and low SCOPA-AUT score groups for mean scores of age (63.7 SD = 8.8 vs. 60.0 SD = 10.1 years, *d* = 0.39, *p* < 0.001), age at symptom onset (61.5 SD = 9.0 vs. 58.1 SD = 10.5 years, *d* = 0.35, *p* < 0.001), age at diagnosis (63.1 SD = 8.8 vs. 59.5 SD = 10.1 years, *d* = 0.38, *p* < 0.001), MDS-UPDRS part I score (7.7 SD = 4.4 vs. 4.0 SD = 2.9, *d* = 0.99, *p* < 0.001), MDS-UPDRS part II score (7.7 SD = 4.5 vs. 4.5 SD = 3.3, *d* = 0.82, *p* < 0.001), MDS-UPDRS part III score (22.4 SD = 8.7 vs. 19.8 SD = 8.8, *d* = 0.30, *p*=0.003), total MDS-UPDRS score (37.8 SD = 13.4 vs. 28.2 SD = 11.3 *d* = 0.77, *p* < 0.001), and S&E scale score (92.5 SD = 5.9 vs. 93.7 SD = 5.8, *d* = 0.21, *p*=0.037). These results suggest that the age and severity of disease based on clinical measures may affect the SCOPA-AUT scores.

A chi-squared test of independence was performed to examine the associations of demographic and clinical variables between the SCOPA-AUT score groups (Table 2). Significant associations were observed in the UPSIT (40.9% vs. 30.0%, OR = 1.61, *p*=0.022), GDS (20.4% vs. 9.0%, OR = 2.58, *p* < 0.001), RBDSQ (37.6% vs. 15.9%, OR = 3.17, *p* < 0.001), ESS (22.7% vs. 10.3%, OR = 2.54, *p*=0.001), QUIP-RS (28.7% vs. 14.7%, OR = 2.35, *p* < 0.001), apathy (5.0% vs. 0.9%, OR = 6.02, *p*=0.013), and fatigue (19.3% vs. 4.7%, OR = 4.82, *p* < 0.001) with the SCOPA-AUT score groups. These results suggest that nonmotor symptoms, such as hyposmia, depression, anxiety, sleep disorders, impulsivity and compulsivity, apathy, and fatigue, may affect SCOPA-AUT scores.

Two logistic regression models were constructed to identify variables that independently predict a SCOPA-AUT score of ≥10. The first model included all significantly associated variables obtained from the previous univariate analyses (Table 3). Patients with probable REM behavior sleep disorder (pbRBD) were 1.86 times more likely to have a SCOPA-AUT score of ≥10 than patients without pbRBD. Increasing age, MDS-UPDRS part I and II scores, and S&E scale scores were associated with an increased likelihood of a SCOPA-AUT score of ≥10. For the second model, the selection of the variables was based on the observation that some of the independent variables evaluating nonmotor symptoms were indirectly evaluated by the MDS-UPDRS (Table 4). Thus, to avoid overlapping of variables, we removed the MDS-UPDRS part I, part II, and total scores. Patients with fatigue (*β* = 4.28, *p* < 0.001), pbRBD (*β* = 2.71, *p* < 0.001), positive QUIP-RS result (*β* = 2.42, *p* < 0.001), and ESS (*β* = 1.88, *p*=0.039) were more likely to have a SCOPA-AUT score of ≥10. Increasing age was also associated with an increased likelihood of a SCOPA-AUT score of ≥10. The results of this second model suggest that age and nonmotor symptoms, such as sleep disorders, fatigue, and impulsivity and compulsivity, best predicted SCOPA-AUT of ≥10 scores in our cohort.

## 4. Discussion

We designed a cross-sectional comparative study to describe dysautonomic symptoms and identify factors associated with autonomic dysfunction in patients with early PD. A proportion of 97% of patients reported the presence of dysautonomic symptoms, with almost 44% reporting a SCOPA-AUT score of ≥10. Urinary symptoms were the most common and frequent dysautonomic symptoms, followed by gastrointestinal symptoms. In addition, patients with early PD and urinary and gastrointestinal symptoms were highly likely to report high SCOPA-AUT scores. The most common symptoms in the cohort were frequent urination, nocturia, and straining when passing stools. Age, clinical measures of severity, and several nonmotor symptoms were significantly associated with SCOPA-AUT scores. The factors that independently predicted high SCOPA-AUT scores in patients with early PD included fatigue, pbRBD, daytime sleepiness, impulsivity and compulsivity, and age.

Autonomic dysfunction is common in early PD. However, studies in patients with early PD are scarce, and the majority of studies that have investigated dysautonomias have focused on gastrointestinal and cardiovascular symptoms. Most studies report gastrointestinal symptoms as the most common dysautonomia, and it is well known that constipation typically presents before the onset of motor symptoms [14, 15]. Urinary symptoms were more prevalent and frequent in our cohort. This finding is in line with the findings of a previous study [16]. We theorize that urinary symptoms are also part of the dysautonomic premotor stage of PD [17]. We observed that straining when passing stools was more commonly reported than constipation and that frequency and nocturia were the most common lower urinary tract symptoms. In addition, our results suggest dyssynergic defecation may be more common than slow transit constipation in PD. The simultaneous presence of lower urinary tract symptoms and lower gastrointestinal symptoms can be explained by the accumulation of Lewy bodies in the nucleus intermediolateralis of the sacral cord and in the motor neurons of the Onufrowicz nucleus early in the disease, as previously reported [18], and proposed as a pathophysiological hypothesis for the development of PD [19]. Further studies aiming to better recognize urinary and gastrointestinal symptoms in early PD may provide an important pathophysiological understanding of the disease.

The factors associated with autonomic dysfunction in our cohort of patients with early PD are in line with those of previous studies, suggesting age, disease severity, and other nonmotor symptoms may affect autonomic dysfunction [12, 20–26] [13, 23, 27]. Our study is the first to report an association between impulsivity and compulsivity and apathy with autonomic dysfunction in patients with early PD.

The factors that significantly predicted high dysautonomic scores, including age, fatigue, sleep disorders, and impulsivity and compulsivity, are similar to previously reported factors in two studies of patients with early PD [24, 28]. These results suggest our findings can be applied to different populations and are consistent in patients with early PD. The present study included PD patients with the shortest disease duration to date examining autonomic dysfunction in PD. Our findings add to previous findings in the literature suggesting a close association between rapid eye movement sleep behavior disorder (RBD) and autonomic dysfunction in patients with early PD in which RBD has been linked to neurodegeneration [29–31]. However, one study reported no association between these variables [32]. More information is required to understand the pathophysiology of fatigue in PD [33]. Our results suggest that a group of patients with PD will present with lower urinary tract symptoms and lower gastrointestinal dysautonomias early in the course of the disease, in addition to fatigue and sleep disorders, and these symptoms will progress with more functional disabilities. This subtype has been previously suggested [6, 16]. Our findings confirm data from previous studies that patients with autonomic dysfunction will develop a more severe PD motor subtype. The other group of patients (i.e., those not showing early autonomic dysfunction) might have another type of nonmotor presentation worthy of studying. We suggest future research studies should focus on better understanding the role of urinary symptoms in premotor stages of the disease.

Some limitations should be considered before interpreting our results. Selection bias was inherently part of our study, since some of the patients in the cohort may progress to multiple system atrophy in subsequent years. Information bias, either recall or reporting, was also part of the study design since scales were used to evaluate outcomes. Stratification of some variables and the use of a multivariate model in the analysis helped reduce the risk of confounding bias.

## 5. Conclusion

Autonomic dysfunction is common in patients with early PD. Lower urinary tract and gastrointestinal symptoms were the most prevalent and frequent dysautonomic symptoms in our cohort. Autonomic symptoms predominated in a group of PD patients early in the disease course and were associated with more severe motor and nonmotor disease. Fatigue, pbRBD, daytime sleepiness, impulsivity and compulsivity, and age independently predicted autonomic dysfunction in early PD.

## Figures and Tables

**Table 1:** Comparison of prevalence and frequency of autonomic symptoms between the high and low SCOPA-AUT groups of patients with PD.

Domains	Prevalence of autonomic symptoms						Frequency of autonomic symptoms					
	Total, *n* = 414 (%)	High SCOPA-AUT (≥10), *n* = 181 (%)	Low SCOPA-AUT (<10), *n* = 233 (%)	*p* value	OR	95% CI of OR	Total, *n* = 414, mean (SD)	High SCOPA-AUT (≥10), *n* = 181, mean (SD)	Low SCOPA-AUT (<10), *n* = 233, mean (SD)	*p* value	*d*	95% CI of *d*
Urinary	392 (94.7)	180 (99.4)	212 (91.0)	<0.001	17.8	2.4 to 133.9	4.2 (3.0)	6.2 (3.1)	2.6 (1.7)	<0.001	1.4	1.3 to 1.7
Gastrointestinal	310 (74.9)	171 (94.5)	139 (59.7)	<0.001	11.6	5.8 to 23.0	2.1 (2.1)	3.5 (2.2)	1.1 (1.2)	<0.001	1.4	1.3 to 1.7
Thermoregulatory	236 (57.0)	144 (79.6)	92 (39.5)	<0.001	6.0	3.8 to 9.3	1.2 (1.4)	1.9 (1.6)	0.6 (0.9)	<0.001	1.0	0.9 to 1.3
Sexual	192 (46.4)	122 (67.4)	70 (30.0)	<0.001	4.8	3.2 to 7.3	1.1 (1.6)	1.8 (1.8)	0.6 (1.0)	<0.001	0.8	0.7 to 1.1
Pupillomotor	141 (34.1)	98 (54.1)	43 (18.5)	<0.001	5.2	3.4 to 8.1	0.4 (0.7)	0.7 (0.7)	0.2 (0.5)	<0.001	0.70	0.5 to 0.9
Cardiovascular	140 (33.8)	87 (48.1)	53 (22.7)	<0.001	3.1	2.1 to 4.8	0.5 (0.8)	0.7 (1.0)	0.3 (0.5)	<0.001	0.6	0.4 to 0.8
Total	403 (97.3)	181 (100)	0 (0)	0.003	18.8	1.1 to 320.6	9.5 (6.2)	14.9 (5.3)	5.4 (2.3)	<0.001	2.3	2.1 to 2.6

SCOPA-AUT: Scales for Outcomes in Parkinson's Disease-Autonomic dysfunction; PD: Parkinson's disease; OR: odds ratio; CI: confidence interval; SD: standard deviation.

**Table 2:** Comparison of demographic and clinical characteristics between the low and high SCOPA-AUT score groups of patients with early PD.

Variable	Total, *n* = 414	High SCOPA-AUT (≥10), *n* = 181	Low SCOPA-AUT (<10), *n* = 233	*p* value	*d* or OR	95% CI of *d* or OR
Age, years (SD)	61.6 (9.7)	63.7 (8.8)	60.0 (10.1)	<0.001	0.39	0.19 to 0.58

Male, *n* (%)	274 (66.2)	116 (64.1)	158 (67.8)	0.427	0.85	0.56 to 1.28

Education, years (SD)	15.6 (3.0)	15.7 (3.0)	15.5 (3.0)	0.598	0.07	−0.13 to 0.26

Hispanic/Latino, *n* (%)	9 (2.2)	5 (2.8)	4 (1.7)	0.473	1.63	0.43 to 6.15

White race, *n* (%)	383 (92.5)	164 (90.6)	219 (94.0)	0.198	0.62	0.30 to 1.29

Family history, *n* (%)	103 (24.9)	44 (24.4)	59 (25.3)	0.838	0.95	0.61 to 1.50

Age at symptom onset, years (SD)	59.7 (10.0)	61.5 (9.0)	58.1 (10.5)	0.001	0.35	0.15 to 0.54

Age at diagnosis, years (SD)	61.1 (9.7)	63.1 (8.8)	59.5 (10.1)	<0.001	0.38	0.18 to 0.57

Disease duration, months (SD)	6.7 (6.6)	7.2 (6.8)	6.3 (6.4)	0.186	0.14	−0.06 to 0.33

Right side at onset, *n* (%)	227 (54.8)	106 (58.6)	121 (51.9)	0.179	1.31	0.88 to 1.94

MDS-UPDRS part I, mean (SD)	5.6 (4.1)	7.7 (4.4)	4.0 (2.9)	<0.001	0.99	0.81 to 1.22

MDS-UPDRS part II, mean (SD)	5.9 (4.2)	7.7 (4.5)	4.5 (3.3)	<0.001	0.81	0.62 to 1.03

MDS-UPDRS part III “off meds,” mean (SD)	20.9 (8.9)	22.4 (8.7)	19.8 (8.8)	0.003	0.30	0.10 to 0.49

MDS-UPDRS total, mean (SD)	32.4 (13.1)	37.8 (13.4)	28.2 (11.3)	<0.001	0.77	0.58 to 0.98

Rigidity, mean (SD)	3.8 (2.6)	4.0 (2.7)	3.6 (2.6)	0.120	0.15	−0.04 to 0.35

Tremor score, mean (SD)	4.4 (3.2)	4.5 (3.2)	4.2 (3.1)	0.363	0.10	−0.10 to 0.29

TD subtype, *n* (%)	294 (71.2)	128 (70.7)	166 (71.6)	0.853	0.96	0.63 to 1.47

HY scale, mean (SD)	1.6 (0.5)	1.6 (0.5)	1.5 (0.5)	0.132	0.20	0.01 to 0.39

S&E scale, mean (SD)	93.1 (5.9)	92.5 (5.9)	93.7 (5.8)	0.037	0.21	−0.40 to −0.01

MoCA <26, *n* (%)	91 (22.1)	42 (23.2)	49 (21.3)	0.645	1.12	0.70 to 1.78

UPSIT <19, *n* (%)	144 (34.8)	74 (40.9)	70 (30.0)	0.022	1.61	1.07 to 2.42

GDS ≥5, *n* (%)	58 (14.0)	37 (20.4)	21 (9.0)	0.001	2.58	1.45 to 4.59

STAI ≥41, *n* (%)	93 (22.5)	48 (26.5)	45 (19.4)	0.087	1.50	0.94 to 2.40

RBDSQ ≥6, *n* (%)	105 (25.4)	68 (37.6)	37 (15.9)	<0.001	3.17	2.00 to 5.03

ESS ≥10, *n* (%)	65 (15.7)	41 (22.7)	24 (10.3)	0.001	2.54	1.47 to 4.39

QUIP-RS any, *n* (%)	86 (20.8)	52 (28.7)	34 (14.7)	<0.001	2.35	1.44 to 3.82

MDS-UPDRS item 1.5 apathy score ≥2, *n* (%)	11 (2.7)	9 (5.0)	2 (0.9)	0.013	6.02	1.28 to 28.21

MDS-UPDRS item 1.13 fatigue score ≥2, *n* (%)	46 (11.1)	35 (19.3)	11 (4.7)	<0.001	4.82	2.37 to 9.79

SCOPA-AUT: Scales for Outcomes in Parkinson's Disease-Autonomic dysfunction; PD: Parkinson's disease; OR: odds ratio; CI: confidence interval; SD: standard deviation; MDS-UPDRS: Movement Disorder Society-Sponsored Revision of the Unified Parkinson's Disease Rating Scale; TD: tremor-dominant; HY: Hoehn and Yahr; S&E: Schwab and England Activities of Daily Living scale; MoCA: Montreal Cognitive Assessment; UPSIT: University of Pennsylvania Smell Identification Test; GDS: Geriatric Depression Scale; STAI: State-Trait Anxiety Inventory—State; RBDSQ: REM Sleep Behavior Screening Questionnaire; ESS: Epworth Sleepiness Scale; QUIP-RS: Questionnaire for Impulsive-Compulsive Disorders in Parkinson's Disease—Rating Scale.

**Table 3:** Regression model predicting a SCOPA-AUT score of ≥10 in patients with early PD.

Variable	Coefficient (*β*)	SE	Exp (*β*)	95% CI of Exp (*β*)	*p* value
Intercept	−10.01	2.40	—	—	—
pbRBD	0.62	0.28	1.86	1.08 to 3.21	0.025
MDS-UPDRS part I score	0.25	0.04	1.29	1.19 to 1.40	<0.001
MDS-UPDRS part II score	0.13	0.04	1.14	1.06 to 1.22	<0.001
Current age	0.04	0.01	1.04	1.02 to 1.07	0.001
S&E scale score	0.05	0.02	1.05	1.01 to 1.10	0.020

SCOPA-AUT: Scales for Outcomes in Parkinson's Disease-Autonomic dysfunction; CI: confidence interval; PD: Parkinson's disease; pbRBD: probable REM Sleep Behavior Disorder; MDS-UPDRS: Movement Disorders Society-Unified Parkinson's Disease Rating Scale; S&E: Schwab and England Activities of Daily Living scale; SE: standard error. The regression model was statistically significant (*p* < 0.001), explained 37.0% of the variance in SCOPA-AUT scores of ≥10, and correctly classified 75.8% of cases.

**Table 4:** Regression model predicting a SCOPA-AUT score of ≥10 in patients with early PD (not including MDS-UPDRS parts I, II, and total).

Variable	Coefficient (*β*)	SE	Exp (*β*)	95% CI of Exp (*β*)	*p* value
Intercept	−4.22	0.79	—	—	—
Fatigue	1.45	0.39	4.28	2.01 to 9.10	<0.001
pbRBD	1.00	0.25	2.71	1.65 to 4.45	<0.001
QUIP-RS	0.88	0.40	2.42	1.41 to 4.14	0.001
ESS	0.63	0.31	1.88	1.03 to 3.44	0.039
Current age	0.05	0.01	1.05	1.02 to 1.07	<0.001
MDS-UPDRS part III	0.02	0.01	1.02	1.00 to 1.05	0.062

SCOPA-AUT: Scales for Outcomes in Parkinson's Disease-Autonomic dysfunction; PD: Parkinson's disease; CI: confidence interval; pbRBD: probable REM Sleep Behavior Disorder; QUIP-RS: Questionnaire for Disorders in Parkinson's Disease—Rating Scale; ESS: Epworth Sleepiness Scale; MDS-UPDRS: Movement Disorder Society-Unified Parkinson's Disease Rating Scale; SE: standard error. The regression model was statistically significant (*p* < 0.001), explained 23.4% of the variance in SCOPA-AUT scores of ≥10, and correctly classified 70.2% of cases.

## Data Availability

Data used in the preparation of this article were obtained from the Parkinson's Progression Markers Initiative (PPMI) database (http://www.ppmi-info.org/data). For up-to-date information on the study, visit http://www.ppmi-info.org.

## References

[1] Obeso J. A., Stamelou M., Goetz C. G. (2017). Past, present, and future of Parkinson’s disease: a special essay on the 200th Anniversary of the Shaking Palsy. *Movement Disorders*.

[2] Magalhães M., Wenning G. K., Daniel S. E., Quinn N. P. (1995). Autonomic dysfunction in pathologically confirmed multiple system atrophy and idiopathic Parkinson’s disease--a retrospective comparison. *Acta Neurologica Scandinavica*.

[3] Cersosimo M. G., Benarroch E. E. (2012). Autonomic involvement in Parkinson’’s disease: pathology, pathophysiology, clinical features and possible peripheral biomarkers. *Journal of the Neurological Sciences*.

[4] Jain S. (2011). Multi-organ autonomic dysfunction in Parkinson disease. *Parkinsonism & Related Disorders*.

[5] Palma J.-A., Kaufmann H. (2014). Autonomic disorders predicting Parkinson’s disease. *Parkinsonism & Related Disorders*.

[6] Van Rooden S. M., Visser M., Verbaan D., Marinus J., Van Hilten J. J. (2009). Patterns of motor and non-motor features in Parkinson’s disease. *Journal of Neurology, Neurosurgery & Psychiatry*.

[7] Merola A., Romagnolo A., Rosso M. (2018). Autonomic dysfunction in Parkinson’s disease: a prospective cohort study. *Movement Disorders*.

[8] Pavy-Le Traon A., Cotterill N., Amarenco G. (2018). clinical rating scales for urinary symptoms in Parkinson disease: critique and recommendations. *Movement Disorders Clinical Practice*.

[9] Visser M., Marinus J., Stiggelbout A. M., Van Hilten J. J. (2004). Assessment of autonomic dysfunction in Parkinson’s disease: the SCOPA-AUT. *Movement Disorders*.

[10] Li Y., Kang W., Yang Q. (2017). Predictive markers for early conversion of iRBD to neurodegenerative synucleinopathy diseases. *Neurology*.

[11] Marek K., Chowdhury S., Siderowf A. (2018). The Parkinson’s progression markers initiative (PPMI) - establishing a PD biomarker cohort. *Annals of Clinical and Translational Neurology*.

[12] Arnao V., Cinturino A., Valentino F. (2015). In patient’s with Parkinson disease, autonomic symptoms are frequent and associated with other non-motor symptoms. *Clinical Autonomic Research*.

[13] Matsubara T., Suzuki K., Fujita H. (2018). Autonomic symptoms correlate with non-autonomic non-motor symptoms and sleep problems in patients with Parkinson’s disease. *European Neurology*.

[14] Asahina M., Mathias C. J., Katagiri A. (2014). Sudomotor and cardiovascular dysfunction in patients with early untreated Parkinson’s disease. *Journal of Parkinson’s Disease*.

[15] Jost W. H. (2010). Gastrointestinal dysfunction in Parkinson’s disease. *Journal of the Neurological Sciences*.

[16] Pagano G., Niccolini F., Yousaf T. (2017). Urinary dysfunction in early de novo patients with Parkinson’s disease. *Movement Disorders*.

[17] Reichmann H. (2017). Premotor diagnosis of Parkinson’s disease. *Neuroscience Bulletin*.

[18] Wakabayashi K., Takahashi H. (1997). Neuropathology of autonomic nervous system in Parkinson’s disease. *European Neurology*.

[19] Sharma A., Kurek J., Morgan J. C., Wakade C., Rao S. S. C. (2018). Constipation in Parkinson’s disease: a nuisance or nuanced answer to the pathophysiological puzzle?. *Current Gastroenterology Reports*.

[20] Awerbuch G. I., Sandyk R. (1992). Autonomic functions in the early stages of Parkinson’s disease. *International Journal of Neuroscience*.

[21] Goldstein D. S. (2006). Orthostatic hypotension as an early finding in Parkinson’s disease. *Clinical Autonomic Research*.

[22] Goldstein D. S., Sewell L., Holmes C. (2010). Association of anosmia with autonomic failure in Parkinson disease. *Neurology*.

[23] Li L., Guo P., Ding D. (2019). Parkinson’s disease with orthostatic hypotension: analyses of clinical characteristics and influencing factors. *Neurological Research*.

[24] Malek N., Lawton M. A., Grosset K. A. (2017). Autonomic dysfunction in early Parkinson’s disease: results from the United Kingdom tracking Parkinson’s study. *Movement Disorders Clinical Practice*.

[25] Van Wamelen D. J., Leta V., Podlewska A. M. (2019). Exploring hyperhidrosis and related thermoregulatory symptoms as a possible clinical identifier for the dysautonomic subtype of Parkinson’s disease. *Journal of Neurology*.

[26] Vetrano D. L., Pisciotta M. S., Lo Monaco M. R. (2015). Association of depressive symptoms with circadian blood pressure alterations in Parkinson’s disease. *Journal of Neurology*.

[27] Merola A., Sawyer R. P., Artusi C. A. (2018). Orthostatic hypotension in Parkinson disease: impact on health care utilization. *Parkinsonism & Related Disorders*.

[28] Stankovic I., Petrovic I., Pekmezovic T. (2019). Longitudinal assessment of autonomic dysfunction in early Parkinson’s disease. *Parkinsonism & Related Disorders*.

[29] Bugalho P., Mendonça M., Lampreia T., Miguel R., Barbosa R., Salavisa M. (2018). Heart rate variability in Parkinson disease and idiopathic REM sleep behavior disorder. *Clinical Autonomic Research*.

[30] Kim J.-S., Park H.-E., Oh Y.-S. (2016). Orthostatic hypotension and cardiac sympathetic denervation in Parkinson disease patients with REM sleep behavioral disorder. *Journal of the Neurological Sciences*.

[31] Postuma R. B., Montplaisir J., Lanfranchi P. (2011). Cardiac autonomic denervation in Parkinson’s disease is linked to REM sleep behavior disorder. *Movement Disorders*.

[32] Leclair-Visonneau L., Magy L., Volteau C. (2018). Heterogeneous pattern of autonomic dysfunction in Parkinson’s disease. *Journal of Neurology*.

[33] Kostic V. S., Tomic A., Jecmenica-Lukic M. (2016). The pathophysiology of fatigue in Parkinson’s disease and its pragmatic management. *Movement Disorders Clinical Practice*.

